# Teacher as role model in developing professional behavior of medical students: a qualitative study

**DOI:** 10.5116/ijme.6443.ae49

**Published:** 2023-04-28

**Authors:** Ova Emilia, Yoyo Suhoyo, Prattama Santoso Utomo

**Affiliations:** 1 Faculty of Medicine, Public Health and Nursing, Universitas Gadjah Mada, Indonesia

**Keywords:** Role model, professionalism, medical teacher, medical education

## Abstract

**Objectives:**

This study aimed to explore students' perspectives on the attributes of medical teachers as role models to students' professional behaviour in the educational process.

**Methods:**

A phenomenological study was conducted to obtain participants' perceptions concerning the professional attributes of medical teachers. The participants were 21 final-year medical students in the School of Medicine, Universitas Gadjah Mada, who had completed and passed the national examination. The participants were recruited purposively to represent genders and performance (i.e., high-performing and average-performing students). The participants were divided into two focus groups based on their performance, each facilitated by non-teaching faculty members to avoid bias. Thematic analysis was conducted to analyze focus group transcripts by two independent coders. Codes were synthesized into themes related to the study aims.

**Results:**

Seven themes were identified related to observed role model attributes, for instance, passionate lecturers, caring and empathetic, supportive and involving, objectivity, incompetence and compromising, poor communication and conflict, and time management. Subsequently, five themes were identified in participants' responses towards the observed role model, for instance, exemplary models, respect and motivating, confusion and inconvenience, avoiding and hate, and value collision and harmonization.

**Conclusions:**

This study revealed a range of role model attributes and responded positively and negatively during learning encounters. As negative attributes are also prominent and observed by students, there is a need for medical schools to perform faculty development for the professional enhancement of medical teachers. Further study should be conducted to investigate the impact of role modelling on learning achievement and future medical practice.

## Introduction

The Indonesian Medical Council, in developing competency standards for Indonesian medical doctors stated that all medical doctors must be professional.^1^ Additionally, it is expected that all medical schools ensure their students receive sufficient education concerning the importance of professionalism. According to several studies, role modelling is the most effective method in teaching professionalism.^2^^-^^4^ This study explores the role of teachers as role models of professional behavior for medical students during their study time.

A role model is defined as someone who is known, has excellence, and is in a position that others aim to achieve or consider as an example.^2^^,^^5^ Role models demonstrate their skills and behavior to be imitated by students consciously or unconsciously,^6^ both positive and negative, and these student experiences can facilitate student learning.^7^ Recent literature shows that there are three main categories that are attributes of role models, which include both positive and negative role model characteristics, namely: the quality of services provided to patients, the quality of teaching conducted, and the personal quality of teachers.^3^^,^^8^ These three categories are then used as learning examples by students.

There are three possible behaviors that can occur in the role modelling process, namely: imitating and being similar to their role model (active identification), having a different attitude from the role model or not wanting to imitate the role model (active rejection), or not experiencing a change in attitude after being together with role models (inactive orientation).^7^^,^^9^ The pattern of rejection and inactive orientation shows that students can choose selectively which ones are alternative models and which ones are anti-models.

Besides having an impact on the development of professionalism, role models are also influential in the formation of professional identity.^5^^,^^10^^,^^11^ Role models influence the formation of students' professional identities by providing positive examples that support their well-being, facilitating reflection and learning, and demonstrating the ability to make decisions based on professional norms, values and attitudes both in medical and non-medical matters. A study in a hierarchical and collectivist culture shows the positive and negative impacts of role modelling on the formation of student professional identity.^11^ Facing a complex learning environment in the clinical setting, students need teachers in the development of their professional identity, especially in providing role modelling, feedback and guidance. Even so, there are times when teachers actually inhibit the process of forming student professional identities by demonstrating negative examples when interacting with patients and even bullying students.

According to the social learning theory described by Bandura,^12^ students learn important professional competencies by observing good role models. In this theory, the behavior demonstrated by the role model will be noticed, remembered, copied/imitated, and even become a motivation and guide for students to act appropriately in the future. Medical schools must facilitate a good role modelling process in order to be able to develop students' professional behavior. Therefore, this study aimed to explore students' perspectives on the attributes of medical teachers as role models to students' professional behaviour in the educational process.

## Methods

### Study Design

A phenomenology study was conducted aiming to explore participants' in-depth perspectives of role model issues during their medical study. The approach applied is appropriate to discover facts related to the studied phenomenon.^13^

### Participants

The study participants were final-year medical students who had completed the preclinical and clinical phases of their study and had passed the national board examination. Participants were in their waiting time for graduation; hence, participants were assured that their participation in this study would not affect their educational outcomes.

A purposive sampling was applied to select a total of 21 participants. Participants' characteristics are described in Table 1. They are all students who accepted the invitation for the focus group discussion among the cohort. The participants were divided into two groups, based on their performance (i.e., high and moderate national examination scores). The sampling strategy ensures the representativeness of the participants within the population.^14^

This study obtained ethical approval from the Medical and Human Research Ethics Committee Universitas Gadjah Mada. Informed consent was obtained from all study participants before participating in this study.

**Table 1.** Participants' characteristics and codes

No.	Code	Gender	Group
01	HP-A-Ma	Male	High Performing
02	HP-B-Ma	Male	High Performing
03	HP-C-Ma	Male	High Performing
04	HP-D-Ma	Male	High Performing
05	HP-E-Fe	Female	High Performing
06	HP-F-Fe	Female	High Performing
07	HP-G-Ma	Male	High Performing
08	HP-H-Fe	Female	High Performing
09	HP-I-Fe	Female	High Performing
10	HP-J-Ma	Male	High Performing
11	AP-A-Ma	Male	Average Performing
12	AP-B-Fe	Female	Average Performing
13	AP-C-Ma	Male	Average Performing
14	AP-D-Ma	Male	Average Performing
15	AP-E-Ma	Male	Average Performing
16	AP-F-Fe	Female	Average Performing
17	AP-G-Fe	Female	Average Performing
18	AP-H-Fe	Female	Average Performing
19	AP-I-Ma	Male	Average Performing
20	AP-J-Fe	Female	Average Performing
21	AP-K-Fe	Female	Average Performing

### Data Collection

Data collection was performed between April – November 2019 in the Faculty of Medicine, Public Health and Nursing Universitas Gadjah Mada. A total of 21 final-year medical students were included in the group interviews. The participants passed the national board examination and were waiting for their conferment. To ensure participants' objectivity and safety, the discussions were guided by moderators who were not involved in participants' clinical education activities. A set of guiding questions were provided to moderate the focus groups (see Appendix). Participants were allocated to two groups of 10 and 11 participants based on their national examination results (high and mid-performing students. The allocation aimed to ensure the homogeneity of participants in each focus group. The group interviews were 90-120 minutes in the Indonesian language. The group interviews were audio-recorded based on the participants' consent. The group interview recordings were then transcribed by professional transcribers.

### Data Analysis

The group discussion transcripts were analyzed using thematic analysis. Initially, two coders conducted open coding of the transcripts independently. Both coders then discussed their coding findings upon the completion of each transcript to overview coding disputes. Intercoder disputes were discussed to achieve agreement. A third coder was appointed when the discussion between the two coders could not achieve consensus.

Member checking to participants was conducted in any skipping voices due to technical problems, and in some ambiguous responses. Codes obtained from the data were then categorized based on professionalism attributes and further synthesized until themes emerged. The researchers ensured data and coder triangulation to minimize bias.^15^ Each chosen transcript was translated into English for reporting and manuscript writing. Participants' details were concealed to ensure safety and anonymity.

## Results

### Students' perspectives of role model attributes during clerkships

A total of 7 themes were identified from the FGDs reflecting participants' perceptions concerning role models during clinical rotation. The themes related to students' observation of medical teachers' attributes which might become an exemplar and role models. The themes were passionate lecturers, caring and empathetic teachers, supportive and involving, objectivity, incompetent and compromising teachers, poor communication and conflict, and effective time management.

### Passionate lecturers

Students perceived that positive role modelling can be seen from both smart and passionate lecturers.

"He (the lecturer) is not only smart, but also humble and passionate towards his scientific interest." (05-HP-E-Fe)

Further exploration revealed that students placed more value on teachers who are passionate in knowledge and humble rather than teachers who are proud based on degree, title or academic rank.

"I admire passionate and humble teachers like him (a professor). As we know, there are lecturers who would be angry when we accidentally write his/her title or degree. But he (a professor) never mind about this issue." (05-HP-E-Fe)

### Caring and empathetic teachers

Students felt safe when they were supervised by teachers who were caring, empathetic and motivating students' success.

"When he (the lecturer) supervised me, he gave many suggestions and passionately taught me. It is not only to pass the examination but also for our future practice. His passion was like a grandfather that wants to see his grandchild successful." (01-HP-A-Ma)

Participants were more comfortable with supervisors who encouraged students to move forward and provided constructive feedback rather than acting as judging and imposing supervisors.

"He (the lecturer) not only examined the proposal I wrote, but also provided feedback, about which parts were okay and that need to be revised. Hence, no hurt feelings at all." (04-HP-D-Ma)

Moreover, students expressed that they were delighted when supervised by kind and patient clinical teachers.

"From my experience, Dr P in K Hospital was very nice. She was good and patient when teaching. I really wish that other clinical teachers will be also like her." (02-HP-B-Ma)

### Supportive and involving

Supervision with patience and passion established students' confidence since they were given responsibility and chances to learn better while they were regarded as a part of the team in patient care.

"The doctor said to the patient, 'Madam, the prescription will be written by the young doctor. He is still learning, please kindly wait patiently.' Then the doctor checked my prescription and confirmed it to the patient." (02-HP-B-Ma)

"The doctor introduced me as his assistant for the patient care. Hence, I felt trusted and became more responsible to the patient." (05-HP-E-Fe)

"He (one of the clinical supervisors) introduced me to the patient, 'He is a clinical student, but soon to be a doctor.' I was humbled to be treated as a fellow colleague." (01-HP-A-Ma)

### Objectivity

Participants emphasized the importance of clinical teachers who consistently work based on applicable standards, including providing fair and objective marks (grades).

"Professional (clinical teachers) is for who works based on standards… For us (students), the objectivity of assessment and marks objectivity is important since it will affect our opportunity for future training/education." (13-AP-C-Ma)

### Incompetent and compromising teachers

Participants perceived negatively the clinical teachers who recommended the use of out-of-date references.

"There was a clinical teacher who, from my point of view, taught obsolete evidence that has been abandoned ages ago. We felt confused since the book cannot be found anymore." (04-HP-D-Ma)

Subsequently, participants complained that some clinical supervisors applied somewhat substandard or even incorrect examination procedures which confused some of the novice students.

"During my internal medicine rotation, there was a clinical teacher who examined the patients carelessly. The skills were definitely different from what we learned from the guidebook. He blamed that the number of the patient is too many to be examined ideally. I think this would provide a bad example for us, as clinical students." (13-AP-C-Ma)

The substandard procedures shown by clinical supervisors might be misleading. As a consequence, the students would have false understanding concerning the procedure.

"I might understand that he (the clinical supervisor) was performing the examination procedure incorrectly. However, other students might wrongly perceive that it was acceptable." (13-AP-C-Ma)

### Poor communication and conflicts

Students highlighted bad relationship and communication among consultants and clinical supervisors which resulted in a non-conducive working atmosphere. The unharmonious relationship was evident in front of the patients, students and other healthcare workers.

"…he (the clinical teacher) said that we (students) should only learn from him since the other consultants are not as good as him. The discussion was done beside the patient, which I think is not good and unprofessional… during the patient consultation, he (the clinical teacher) also told the nurse that the treatment of the other consultant was wrong. The discussion occurred beside the patient. We (students) understand that the previous treatment was incorrect, but it is not necessary to blame the consultant in front of the patient." (05-HP-E-Fe)

Participants complained that disputes between clinical supervisors inflicted conflicts of interest. Students felt they were confused and pressed between supervisors. Hence, students' learning process was seriously affected.

"We were obliged to present at his (a clinical teacher) outpatient clinic every day when we wanted to get an A mark. However, we should also attend other teachers' clinics or ward rounds. This conflicting interest made us confused and stressed." (16-AP-F-Fe)

The negative learning atmosphere due to conflicts between supervisors was regretted by the students. Although the teaching hospital has many interesting cases, students could not learn appropriately due to the bad relationships between consultants.

"I really felt uncomfortable. It appears that weeks of my internal medicine clinical placement in K hospital was useless since the consultants were in conflict continuously." (05-HP-E-Fe)

### Effective time management

Lecturers are also expected to be consistent with applicable rules, particularly discipline and timely responsiveness.

"Let's say that students are obliged to be tidy, disciplined and ontime. The same rules should also be applied and obliged by the lecturers. When we are expected to respond to tasks immediately, please do respond to our questions timely." (11-AP-A-Ma)

"…we respect clinical teachers who were diligent, on-time and responsive to our questions. So it is not only students who should be disciplined, but also so should the lecturers and teachers." (13-AP-C-Ma)

Students raised a concern about clinical supervisors who were racist and discussed aspects irrelevant to learning, as bad role models.

"…and I realized that dr. X (a lecturer) dislikes Malaysian students. He scolds Malaysian students in every one of his lecture sessions" (08-HP-H-Fe)

Participants highlighted clinical supervisors and department administrators who suspended grades which were perceived as an intention to make students' tasks more complicated.

"Our friends have completed their rotation in the ophthalmology department for two months, but the clinical supervisor had not completed their grading…From my point of view, the administrator should immediately compile and complete our grades. Sometimes, our mark was suspended due to a single signature missing." (01-HP-A-Ma)

Students were confused due to unclear consultation and coordination flow to which they needed to adhere. Inconsistent working and coordination flow, including consultation, confused students during clinical learning and patient care.

"As student doctors, we were confused most of the time when dealing with patients. We felt confused about whom we should consult. When we asked the residents, they responded 'We are very busy'; When we asked the consultants, they responded 'Have you consulted the residents?' Some departments do have a clear coordination flow, but many others do not." (13-AP-C-Ma)

### Responses towards role model behaviors

A total of 5 themes were identified on participants responses towards the observed role model behaviors. For instance, exemplary models, respect and motivating, confusion and inconvenience, avoiding and hate, and value collision and harmonization.

### Exemplary models

Students responded to good role model behaviors and set them as a good examples to learn.

"When I saw him (a clinical teacher) managing the patients, I wanted to learn more and make him as a role model. A teacher like him should be an example for other teachers" (19-AP-I-Ma)

"I learned an excellent patient management from dr. E in U Hospital. Most of his patients leave the consultation room with happy feeling, as if their illness is cured." (15-AP-E-Ma)

Many participants felt inspired and motivated to pursue a similar career as the role model.

"Every time I saw lecturers who behave professionally, I became inspired. This is the doctor that I want to be!" (15-AP-E-Ma)

"Yes, the consultant emitted positive influence for us, we would become positively inspired." (01-HP-A-Ma)

### Respect and motivating

Some students elaborated that respect was built toward lecturers or clinical teachers who showed positive behaviors.

"I really liked consultants who showed good empathy… the doctor was patiently listening to the complaints. I respect him so much since we seldomly find doctors with that attitude" (17-AP-G-Fe)

Good role modelling and behaviors shown by the lecturers and clinical teachers drive students' learning motivation.

"She (a clinical teacher) always made me excited to learn everyday. When she came to the ward, I suddenly became enthusiastic to learn although I was tired." (02-HP-B-Ma)

"His (a consultant) professional examples drive all of us to learn and advance, to prepare ourselves as good doctors." (16-AP-F-Fe)

"I was disappointed to be assigned to S Hospital in the beginning. However, the clinical supervisor provided a positive learning atmosphere for us. Ultimately, I enjoyed all of the learning processes in S Hospital." (01-HP-A-Ma)

### Confusion and inconvenience

Participants also expressed confusion when the teachers performed malicious, unpleasant, or negative behavior.

"I was really shocked and confused since the clinical supervisor was overly touchy to female students." (21-AP-K-Fe)

"…as I perceive from dr. F, he is very brilliant. However, he is often rude in his words. This combination is rather questionable for me…" (11-AP-A-Ma)

Inconvenience often occurs when students experienced or saw negative role modelling shown by lecturers and clinical teachers.

"The conflicts between consultants were a shame for us because the situation became rather awkward and inconvenient." (05-HP-E-Fe)

Some students experienced hate as a response to negative role model behaviors. The extent of hatred might start from badmouthing.

"…we were always complaining to each other after a session with the lecturer, we tried to compare her with other lecturers and even badmouthing…" (05-HP-E-Fe)

### Avoiding and hate

Participants reported that some of them attempted to avoid teachers with malicious behavior. Some participants also ignored the clinical teachers with bad habits.

"Finally, I concluded that this kind of behavior should be avoided. I shouldn't learn from him (a clinical teacher) since his attitude is hateful! I don't want to be like that." (05-HP-E-Fe)

"I fully avoided the consultant since he was discriminatory. I also avoided activities with Dr A since his attitude confronted my values." (20-AP-J-Fe)

"I would ignore him (a clinical teacher). If he dismissed me, I would just go on… From my point of view, his attitude is already ingrained. So, why should we bother?" (01-HP-A-Ma)

Participants also responded passively by maintaining their patience. This passive response was particularly reported when the teacher's negative behavior was persistent.

"We tried to compare his (clinical supervisor) behavior to other groups and found that it was consistent…. We have to be patient." (03-HP-C-Ma)

In addition, unpleasant responses such as disappointment and anger were reported by some participants.

"He (clinical supervisor) was discriminating against us since we are from UGM. I was deeply disappointed, and it made me angry." (01-HP-A-Ma)

Despite the negative behavior shown by lecturers and clinical supervisors, students stayed pragmatic during their encounters.

"(when facing difficult clinical supervisor)…I would just attempt for minimum efforts to meet the administrative requirements for passing the rotation." (03-HP-C-Ma)

### Value collisions and harmonization

Participants perceived that the responses towards the clinical teachers' behaviors were related to their own personal values. Some behaviors were pertinent to students' values as responded positively by the students.

"We were often being scolded by dr. B when we only focused on the medical aspects during patient encounters. He reminded us that patients should not be treated as experimental subjects. This value, for me, is really a quality of a good role model." (03-HP-C-Ma)

Conversely, some unethical behaviors of clinical teachers would collide and conflict with students' values responded negatively by the students.

"I felt disturbed with his (a clinical teacher) attitude. So, I clearly declared myself to avoid him totally. There is nothing to be learnt from him since his attitude is fully opposite of my conscience and values." (20-AP-J-Fe)

Participants' observations and responses varied towards different role model attributes of medical teachers.

## Discussion

This study shows a range of behavior of preclinical and clinical teachers observed by students. The themes showed both positive and negative role model attributes observed by the students. The positive role model aspects were discipline, effective communication, objective feedback, empathy and caring. On the other hand, the observed negative role model aspects that should be avoided included out-of-date teaching, compromising patient safety, unhealthy competitive behavior and conflicts of interest. This study also found various responses to positive role models, such as respect, admiration, inspiration and willingness to learn. In contrast, negative role models were responded to by complaints, confusion, hate, avoidance and pragmatism.

Positive role modelling from teachers may encourage students to think and act more creatively, allowing them to also step outside their comfort zone to participate in patient care while continuously developing themselves. It is important to achieve competencies and develop their professional identities as a doctor.^8^^,^^10^^,^^11^ Together with good mentorship, emphasizing obeying the code of conduct, and the concept of a good doctor, this positive role modelling will influence on professional behaviour of students.^10^ This finding is relevant to experiential learning principles where teachers should provide effective support to their students, as reported by Dornan.^16^ Moreover, teachers with positive attitudes may establish a safe and convenient learning environment for students to learn optimally in clinical settings.^17^ Nevertheless, teachers' expertise in their speciality also plays an essential role in the success of clinical teaching (e.g., the right person doing it), as suggested by Harden.^18^

Positive role modelling found in this study should be promoted and enhanced. Positive role model is one of the roles of a medical teacher, in which such good attitudes should be shown to students.^19^ Burgess and colleagues^20^ reported that teaching and facilitation skills are perceived as admirable characteristics of clinical teachers. This concept is also relevant to Indonesia's educational philosophy proposed by Indonesia's father of education, Ki Hajar Dewantara, named 'Ing Madya Mangun Karsa'. The statement means teachers should not only be active in transferring knowledge but also facilitating learning, nurturing good characters and developing a sense of independence in the students.^21^ Teachers are expected to guide students to develop their role stages from initially as passive observers to being active observers, then through actor rehearsal to finally becoming actors in the real-life performance.^16^^, ^^22^

During the FGDs of this study, students tended to report more negative characteristics of the teachers than positive aspects. The negative experiences might be easier to recall. Teachers should be aware of this trend and carefully behave since students might observe and even consider the teachers' negative behavior as an appropriate role model. The negative role modelling will inhibit the formation of students' professional identities.^11^ Reflection should be performed adequately to ensure students understand what aspects they should perform or avoid.^23^ A survey on medical students reported that 62% of students tended to report observed malicious behaviors, which might contribute to an increased risk of students' performing the negative behavior later.^23^ Negative examples might be useful if teachers conduct adequate reflection and debriefing in all of the learning encounters. Teachers should facilitate and supervise learners closely prior to, during and after every encounter, particularly during the reflection session.^24^

This study found that poor teamwork, ineffective communication, bullying and teachers' self-interest resulted in students' learning discomfort. This finding indicates that negative behaviors reflect the failure of teachers to create good role modelling and academic atmospheres. Medical teachers might encounter this condition due to a lack of facilitation experience, less updated knowledge or information, being overly busy and/or exhaustion/burn-out.^25^ Consequently, learning quality and outcomes might be compromised. Medical schools should conduct robust faculty development and coaching activities for clinical teachers to improve their facilitation skills.

Students were disappointed concerning teachers who teach with out-of-date materials. The students expect to learn from the latest evidence. Medical teachers should teach students with the best and current evidence to ensure optimal patient care. This up-to-date resourcefulness is an obligation of teachers as a modern learning source.^18^ Providing the current best evidence and materials is also considered a good role model behavior of clinical teachers. Good role modelling should include personal aspects, teaching skills and clinical attributes. Students might be underserved or even done a disservice if any of the aspects were not effective.^25^

This study revealed a range of responses towards negative role models, including confusion, inconvenience, hate, avoidance and ignorance, patience, disappointment and anger, pragmatism, and value collision-harmonization. Acute responses (i.e., confusion, hate, inconvenience, anger, and disappointment) normally occur immediately after an encounter with a bad experience. However, chronic and critical responses (i.e., avoidance, value collision and harmonization) require in-depth reflection and quality feedback to ensure reflective learning.^26^ Reflection directs bad experiences into learning opportunities. Educational institutions should regard both professional and humanistic attributes during role modelling facilitation and reflection.^27^

This research pointed out that role modelling was not only related to the teachers' behavior, but also involved the students' responses towards the role model. Students' coping mechanisms are essential to ensure that effective role modelling is applied for quality learning. Educational institutions should prepare and equip students with the capability to avoid dysfunctional coping mechanisms.^28^ The appropriate and socially acceptable coping mechanisms are not only useful during education, but also for later professional development.

### Limitations

This research has revealed a range of role model attributes of medical teachers, as also students' responses toward the role model in an institution. The generalisability of the outcomes of this study needs to be confirmed using research in a wider scope. The study was conducted in a cohort of students and might need to be corroborated with longitudinal or multi-year observations. Further investigation concerning the impact of role modelling towards learning outcomes and future practice should also be investigated.

## Conclusions

This study revealed that students viewed a range of role model attributes and responded both positively and negatively during learning encounters. As negative attributes are also prominent and observed by students, there is a need for medical schools to perform faculty development for the professional enhancement of medical teachers. In addition, students' responses concerning the observed behaviours vary and need to be facilitated with thorough reflection. Medical schools should prepare both teachers and students to ensure good role modelling and robust reflection on good role models. Further study should be conducted to investigate the impact of role modelling on learning achievement and future medical practice.

### Conflict of Interest

The authors declare that they have no conflict of interest.

## Supplementary material

Supplementary file 1 Appendix. Focus group guiding questions
