# Supplementary material for: Teacher as role model in developing professional behavior of medical students: a qualitative study
Source: Int J Med Educ. 2023 Apr 28;14:55–62. doi: 10.5116/ijme.6443.ae49 (PMC10693392; doi:10.5116/ijme.6443.ae49)
Supplement: Supplementary file 1 — Appendix. Focus group guiding questions [file ijme-14-55-S1.pdf]

Appendix A

Overview of the non-technical skills observation system (behavioural markers are examples and therefore incomplete)

No	Guiding Questions	
	Professional	Unprofessional
1	How do you describe a professional medical teacher?<br>Probe: Do you have examples of professional medical teachers who possess your description before?	How do you describe an unprofessional medical teacher?<br>Probe: Do you have examples of unprofessional medical teachers who possess your description before?
2	How was your feeling when you encountered a professional medical teacher? Please explain in detail.	How was your feeling when you encountered an unprofessional medical teacher? Please explain in detail.
3	What were your responses when you encountered a professional medical teacher?<br>Probes:<br>• How did you cope personally with his/her professional behavior?<br>• How did you see others respond to his/her professional behavior?<br>• Did you consider to follow his/her behavior? Please explain your reason.	What were your responses when you encountered an unprofessional medical teacher?<br>Probes:<br>• How did you cope personally with his/her unprofessional behavior?<br>• How did you see others respond to his/her unprofessional behavior?<br>• Did you consider to avoid his/her behavior? Please explain your reason.
4	Were there any faculty/lecturer who taught you on how to behave professionally, excluding bioethic and medicolegal lessons?<br>Probes:<br>• When was the lesson that happened (e.g., during rounds, clinical reflection, tutorials, etc)?<br>• What aspects did your lecturer/teacher stress on?	
5	What are your suggestions to the medical school to prepare graduates to behave professionally?	
